# Automated Raman Micro-Spectroscopy of Epithelial Cell Nuclei for High-Throughput Classification

**DOI:** 10.3390/cancers13194767

**Published:** 2021-09-24

**Authors:** Kevin O’Dwyer, Katarina Domijan, Adam Dignam, Marion Butler, Bryan M. Hennelly

**Affiliations:** 1 Department of Electronic Engineering, Maynooth University, Maynooth, Ireland; kevin.odwyer@mu.ie; 2 Department of Mathematics and Statistics, Maynooth University, Maynooth, Ireland; katarina.domijan@mu.ie; 3 Department of Biology, Maynooth University, Maynooth, Ireland; adam.dignam@mu.ie (A.D.); marion.butler@mu.ie (M.B.); 4 Department of Computer Science, Maynooth University, Maynooth, Ireland

**Keywords:** Raman spectroscopy, automated cytology, high-throughput classification, cellular classification, screening, ThinPrep

## Abstract

**Simple Summary:**

We demonstrate an automated Raman cytology system designed for high-throughput and reproducibility. The system uses a Raman spectroscopy system integrated into a conventional microscope, all controlled electronically via and open source software, Micro-Manager. The system can automatically identify and probe epithelial cell nuclei for Raman spectroscopy. 6426 HT1197 (high-grade bladder cancer) cell spectra, and 7499 RT112 (low-grade bladdercancer) cell spectra were recorded. The data was subsequently culled and processed for denoising and artifact removal. We demonstrate, using multivariate statistical analysis, that the cells can be distinguished, using a variety of approaches with accuracy, sensitivity and specificity in excess of 95%.

**Abstract:**

Raman micro-spectroscopy is a powerful technique for the identification and classification of cancer cells and tissues. In recent years, the application of Raman spectroscopy to detect bladder, cervical, and oral cytological samples has been reported to have an accuracy greater than that of standard pathology. However, despite being entirely non-invasive and relatively inexpensive, the slow recording time, and lack of reproducibility have prevented the clinical adoption of the technology. Here, we present an automated Raman cytology system that can facilitate high-throughput screening and improve reproducibility. The proposed system is designed to be integrated directly into the standard pathology clinic, taking into account their methodologies and consumables. The system employs image processing algorithms and integrated hardware/software architectures in order to achieve automation and is tested using the ThinPrep standard, including the use of glass slides, and a number of bladder cancer cell lines. The entire automation process is implemented, using the open source Micro-Manager platform and is made freely available. We believe that this code can be readily integrated into existing commercial Raman micro-spectrometers.

## 1. Introduction

Raman micro-spectroscopy (RMS) is a powerful technique for the identification, classification, and diagnosis of cancer cells and tissues [1,2,3,4,5,6,7,8,9]. The prefix “micro” indicates integration of a Raman spectroscopy (RS) system into a microscope such that spectra are obtained from sub-micron areas in a sample. RS is based on inelastic light–matter scattering, whereby the scattered photons have lost energy relative to the incident photons equivalent to the energy of certain Raman-active molecular bonds present within the sample. RS can identify biomolecular changes within cells as they progress from a healthy to a cancerous state [1,2,3]. The magnitude of the frequency shift is dependent on the molecular structure of the sample. For a complex mixture of different chemicals, such as in a cell, a highly unique Raman spectrum can be observed that is sometimes described as a ‘fingerprint’. Multivariate statistical analysis is applied to Raman spectra for classification, whereby statistical pattern recognition algorithms, such as principal components analysis (PCA) combined with linear discriminant analysis (LDA), are applied to identify subtle changes across datasets that can be used to accurately differentiate between different pathological groups [1,2,3,4,5,6,7,8].

Malignancies of epithelial tissue, or carcinoma, account for 80–90% of all cancer cases [10], and clinical cytology—whereby oral, cervical, or bladder epithelial cells are visually inspected—is routinely applied in pathology labs for diagnostics. Distortion of the cell nucleus is common for carcinoma, and pathologists are trained to identify pertinent morphological features. The applications of RMS to clinical cytology with specific targeting of the cell nucleus for classification has shown promising results with many reporting the classification of bladder [3,4,5,6], cervical [7,11,12,13], and oral [8,14] cytological samples with an accuracy that is significantly greater than standard pathology. However, issues around reproducibility and speed have thus far hindered the clinical adoption of Raman cytology into the clinic. In this paper, we focus on the development of an automated RMS system for the classification of epithelial cells, based on the biochemical composition of the nucleus, with the ultimate goal of high-throughput, non-invasive screening.

This paper builds on recent work in the development of robust Raman cytology platforms that are fully automated and high-speed. Schie et al. have published several papers that describe a high-throughput automated Raman cytology platform with several applications [15,16,17,18,19,20,21]. The basic platform [15,16] has several important features, including a defocused laser spot to excite a large cell area with high power, thereby facilitating shorter exposure times, and a LabVIEW-based automaton process, which identifies cell position using image processing and controls a translation stage to align the cells with the laser spot. The system employs a 400 mW laser with a wavelength of 785 nm focused to an area of 10 μm${}^{2}$, which enables acquisition times of <1 s for various cell types. The image processing component is central to the overall system. This is based on several steps, which, in essence, identify the cell position as the center of the bulk cell area, appearing dark with respect to the bright-field background. Combined with multivariate statistical classification, the system was demonstrated to automatically classify hundreds of thousands of different cells based on their Raman spectra in relatively short times. In its first application, the system was successfully applied to various white blood cells as well as pancreatic cells [15,16] with a maximum rate of 5000 cells/h. Drug testing is a key application area for automated Raman cytology systems. In this capacity, the same system was applied by the authors to (i) study the effect of dithiothreitol on the diatom *Phaeodactylum tricornutum* [17]; (ii) to analyse multi-well plates [18] for the purpose of developing a Raman-based cell viability assay; specifically, on the effect of doxorubicin concentration on monocytic THP-1 cells; and (iii) to investigate the effect of the targeted cancer drug panitumumab on colorectal cancer cell lines [20].

Although not a target application of the system proposed here, the study of pathogens, and in particular, bacteria, has been another key application area of automated Raman cytology systems in recent years. The automated system described in the previous paragraph was adapted to record spectra of single isolated neutrophils from human peripheral blood [19], which were stimulated via an in vitro infection model with heat-inactivated bacterial and fungi pathogens; the system captured 20,000 neutrophil spectra across various treatment groups, originating from three donors. Another system developed by Douet et al. [22] was designed to provide automated Raman spectroscopy of individual bacteria cells; once again, this automated system is based on image processing in order to automatically identify the bacterial cell position, followed by alignment of the cell with the excitation laser. In this case, the image processing component relies on the availability of an out-of-focus diffraction pattern facilitated by the use of a spatially coherent illumination source. The recorded image can be described as an in-line digital hologram, which can be subjected to numerical propagation [23] in order to obtain an in-focus image of the sample. The cell position can be identified based only on image contrast, whereby the bacterial cell appears dark against a bright background.

In this paper, we present an automated Raman cytology system with several contributions: (1) This system utilises a simpler image processing component than previous systems, which is based on a single step. It is shown that this approach can accurately identify the epithelial cell nucleus position, which, to the best of our knowledge, has not been a target area for previous automated systems. (2) The system can be applied to unlabelled ‘phase-only’ adherent cells, which produce low image contrast. (3) The system is demonstrated to work with the ThinPrep standard [5,7,12,24], an established instrument and protocol used to prepare cytology samples in hospital settings, such as the cervical ‘Pap’ smear. (4) Finally, the method is based around the open-source Micro-Manager platform [25], which is freely available. The associated code is supplied in an online repository [26] and is described in detail in the supplementary information. This approach can be implemented easily on any existing RMS system that uses a motorised translation stage and a computer-controllable microscope lamp and excitation laser, which are commonplace in modern life-science microscopes and commercial RMS systems.

## 2. Automation

### 2.1. Principle of Automation: Identifying Cell Nucleus Position Using the Nucleus ‘Microlens-Effect’

Central to the proposed automated Raman cytology system is an image capture and processing methodology that facilitates the rapid identification of cell nuclei, which can subsequently be targeted for RS. The cell nucleus, which contains almost all of the cell DNA, is the primary target for Raman-based classification of the epithelial cell type; in some studies, the nucleus is targeted at several different points and the resulting spectra are averaged, while in other studies, this ‘averaging’ is achieved optically by using a relatively large laser spot for excitation [1,2,3,4,5,6,7,8,9,11,12,13,14]. However, this approach is complicated by the difficulty in clearly identifying cellular features, such as the cell nucleus, using bright-field microscopy. Adherent epithelial cells are commonly described as weakly scattering phase-only objects that appear almost transparent when imaged using bright-field microscopy. Although several imaging modalities exist to improve the image contrast of such objects, such as phase-contrast and differential interference contrast, these methods cannot reliably be used to identify the cell nucleus. Furthermore, these modalities require dedicated equipment, such as a phase-contrast objective (which includes an annular filter) or polarising optics, which are preferably avoided when using RS. Fluorescence microscopy is the gold standard for identification of the cell nucleus, whereby a fluorescent stain, such as 4’,6-diamidino-2-phenylindole (DAPI), which can penetrate the cell membrane and bind to adenine–thymine-rich regions in DNA. However, combining fluorescent microscopy and RMS is not straightforward since both methods require dedicated excitation sources and different optical collection systems and filters behind the microscope objective. The fluorescence spectrum can also overlap with the Raman spectrum, although it is possible to ensure that the two bands are mutually exclusive [27]. A further complication is that, unlike RS, fluorescent labels are commonly incompatible with living cells [28].

In this paper, we utilise the ‘microlens-effect’ of the cell nucleus in order to identify its position, whereby the phase-delay introduced by the cell nucleus has the effect of focusing partially-coherent illumination, similar to the effect of a micro-lens. This phenomenon has previously been investigated for the purpose of cell counting [29], which was the inspiration for this paper. In that study, human neuroblastoma cells were imaged in culture, using a low magnification objective in order to capture a large number of cells in the field of view. The authors demonstrated that a green filter and a 130 μm pinhole placed immediately above the culture flask generated bright-spots in a defocused plane ≈50 μm from the object plane, which were approximately centred in the fluorescing regions (nuclei) of the cells. This was confirmed, using the green fluorescent protein-tagged nuclear histone H2B protein.

In the system proposed in this paper, partially coherent illumination is generated, using only the microscope components that would be found in an existing RMS system. Such illumination can easily be obtained by closing the condenser aperture diaphragm such that the spatial coherence of the Kohler-illumination is maximised. We also make no attempt to apply a colour filter to the tungsten halogen lamp in order to enhance the bright-spot contrast. Another important difference in our work with respect to [29] is that the approach taken here makes use of a high magnification/numerical aperture microscope objective, which can resolve several bright spots in the ‘focal-plane’ of the cell nucleus corresponding to various sub-cellular features. Even with this simplified approach, we demonstrate that it is possible to identify the cell nucleus position with a sufficient degree of accuracy for subsequent targeting with RMS.

**Figure 1:**
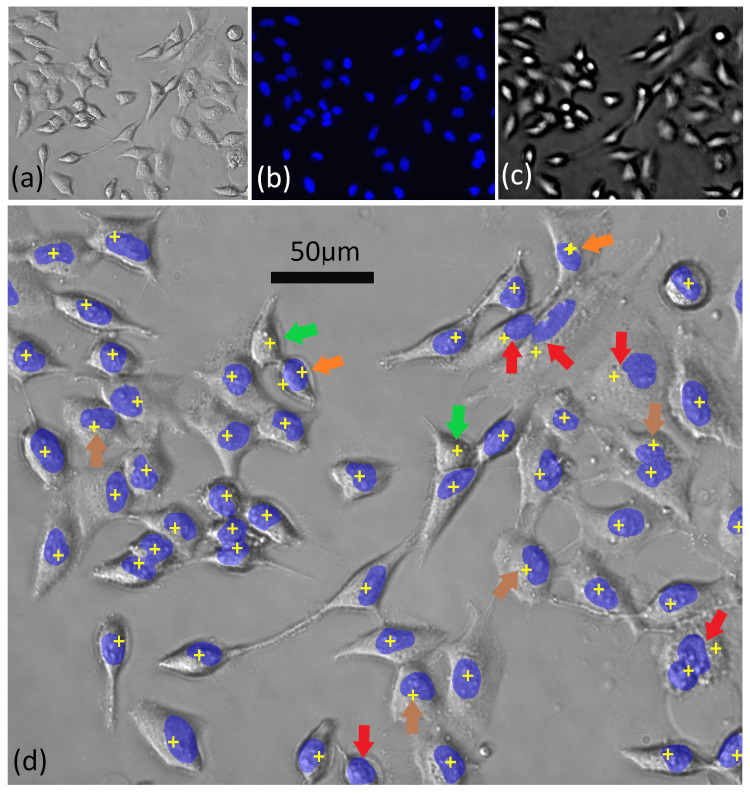
Live HeLa cells in medium: (**a**) bright-field image; (**b**) fluorescence image using DAPI; (**c**) bright-spot image: sample at 50 μm from focal plane; (**d**) combined images. Images (**a**,**b**) are superimposed together with the positions of the local maxima in image (**c**) following image processing. The arrows are described in the text.

**Figure 2:**
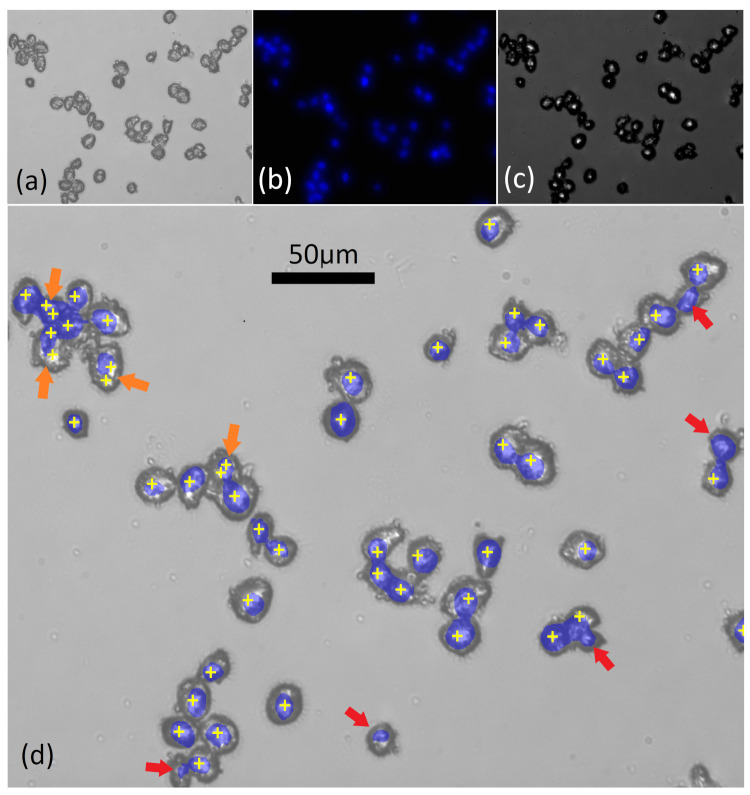
HeLa cells on ThinPrep slides: (**a**) bright-field image; (**b**) fluorescence image using DAPI; (**c**) bright-spot image: sample at 14 μm from focal plane; (**d**) combined images. Images (**a**,**b**) are superimposed together with the positions of the local maxima in image (**c**) following image processing. The arrows are described in the text.

The applicability of this method of nuclear targeting is demonstrated for two forms of sample preparation: (1) live adherent epithelial cells in medium as shown in Figure 1, and (2) the same cells prepared using the ThinPrep standard as shown in Figure 2. ThinPrep is a clinical standard for the preparation of cyto-histological samples, particularly in the areas of cervical screening and urine cytology. The ThinPrep standard, including the use of associated fixatives and glass slides, was previously shown to be compatible with Raman micro-spectroscopy [5,7,12,24,30]. HeLa cells were selected for this initial investigation, due to their well known morphology and were prepared as described in Section 3.1.1 and imaged using an IX81 fluorescence microscope as described in Section 3.2. In Figure 1a, the live cells are shown in medium. The image was slightly defocused by displacing the sample approximately 1μm from the focal plane, and oblique illumination was used in order to improve the visualisation of the cell boundaries. In Figure 1b, the corresponding fluorescence image, using DAPI is shown, which highlights the nucleus in each cell. In Figure 1c, the bright-spot image is shown. This was obtained by moving the sample up a distance of 50 μm from the focal plane, which was found to provide the highest contrast image, although larger displacements could still be used to successfully resolve the bright spots. In this case, axial illumination is used to ensure that the position of the bright-spot aligns with the nucleus of the cells when positioned in the image plane; in Figure 1d, the information from these three images is combined. Figure 1a,b are superimposed together with the positions of the local maxima in Figure 1c, which are shown as yellow targets. The red arrows highlight nuclei that are just missed by the target. The green arrows highlight cells that appear to be correctly targeted but did not fluoresce. The brown arrow highlights cases that are just at the edge of the nucleus but which would likely provide meaningful Raman spectra for a laser spot size of >1 μm. Finally, the orange arrows highlight cell nuclei that are double targeted. Based on an analysis of several such images, we estimate successful (single) targeting of >75% of HeLa cell nuclei.

A similar analysis was applied to samples prepared, using the ThinPrep standard. A bright field image of these cells is shown in Figure 2a. The cells have a thicker morphology and a greater depth of field than for the adherent case, making it more difficult to record an in-focus image with a high NA objective. In Figure 2b, the corresponding fluorescence image using DAPI is shown, once again highlighting the nucleus in each cell. It is clear that the nuclei for the ThinPrep case are smaller in area than those for the adherent case. In Figure 2c, the bright-spot image is shown. This was obtained by moving the sample up a distance of 14 μm from the focal plane, which was found to provide optimal contrast. Interestingly, the ‘focal-length’ of the ThinPrep HeLa cells (i.e., the axial displacement providing the highest bright-spot image contrast) is significantly shorter than for the case of adherent cells, owing to their thicker, rounder morphology. In Figure 2d, the information from these three images is combined. Figure 2a,b are superimposed together with the positions of the local maxima in Figure 2c following Gaussian filtering, which are shown as yellow targets. The red arrows highlight nuclei that are just missed by the target, and the orange arrow highlight cases that are double targeted. Based on an analysis of several such images, we estimate successful (single) targeting of >85% of HeLa cell nuclei prepared using the ThinPrep standard.

In this section, we have demonstrated that it is possible to identify cell nucleus position, using a standard bright-field microscope with a closed condenser aperture, both for adherent live cell and also for cells prepared using the ThinPrep standard. In the next section, we outline a global automation routine for Raman cytology that makes use of this. Although in subsequent sections we focus our results on ThinPrep samples due to their clinical relevance, we have also found that adherent cells work equally well with this automated routine.

### 2.2. Global Automation Process

**Figure 3:**
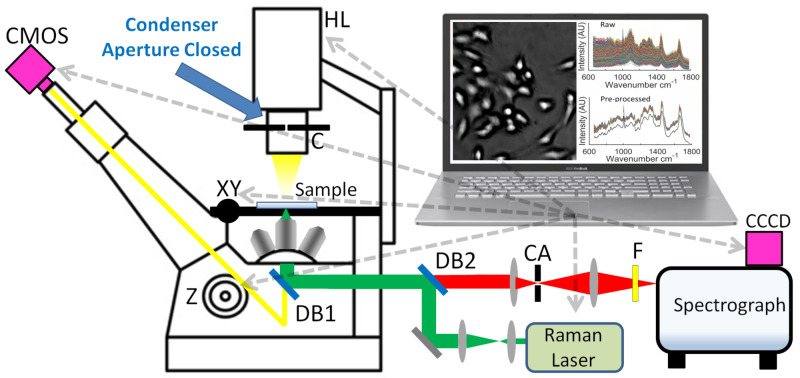
Schematic of opto-electronic system. The computer controls several elements in the system, using an open-source integrated hardware control and image processing system: Micro-Manager. The various components, and symbols, are described in Section 3.3.

The nucleus ‘microlens-effect’ is used as a basis for targeting of cells for an automated RMS platform. The system uses a conventional Olympus-IX81 microscope, controlled with a PC via the IX2-UCB control box, which allows for electronic control of the microscope objective focus position (Z-stage) and the white-light lamp. As illustrated in Figure 3, the open-source Micro-Manager software system [25] can be used to control the IX2-UCB, as well as several other opto-electronic components in the system, including an inexpensive CMOS digital camera inserted into the eyepiece of the microscope, the XY-translation stage, the laser used for Raman excitation, and the cooled CCD detector used to capture the Raman spectrum, all of which are described in more detail in Section 3.3. The Micro-Manager platform allows for the automatic control of these components with a modified Beanshell scripting interface, which also facilitates the use of the ImageJ library for image processing [31,32]. The entirety of the automation platform for collection of the cell spectra is written using this scripting interface, and these scripts are freely provided in an online repository [26]. A key feature of this automation system is that the condenser aperture is closed to a minimum in order to maximise the contrast in the bright-spot image as described in the previous section.

**Figure 4:**
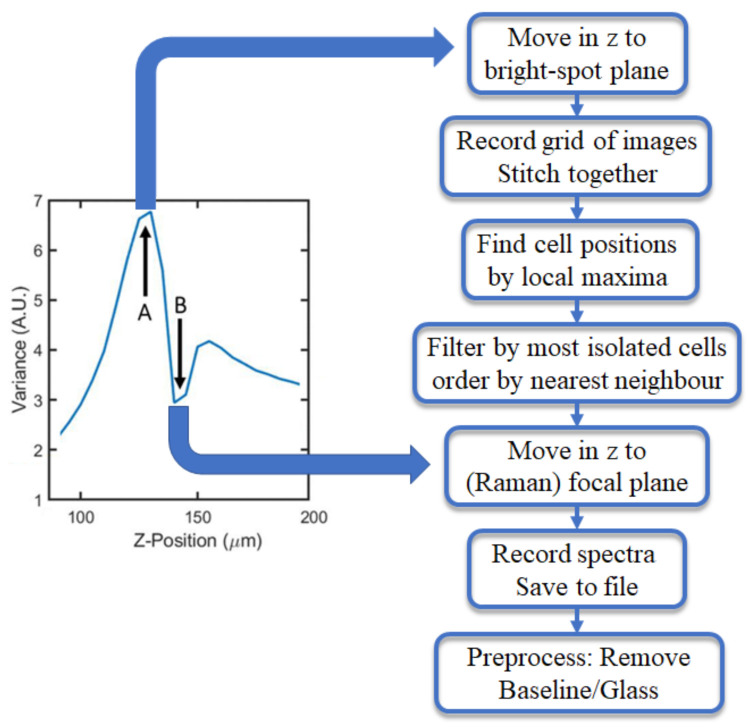
Variance profile shown on left side with points A and B corresponding to the sample positioned at the bright-spot plane and the focal plane of the microscope, respectively—corresponding to images shown in Figure 2a,c. On the right side, a high-level flow chart is shown for the overall automated Raman cytology process, which includes moving the sample between these two planes.

In order to locate the cells, the Z-position of the bright-spot plane must first be determined. However, as described in the previous section, the position of this plane will vary significantly depending on cell morphology. Therefore, in order to account for different cell morphologies, this plane is found, using an auto-focusing routine. A series of spatially coherent light images are recorded across a range of focal positions in the same field-of-view (FOV). It is found that when the variance of a given FOV is maximised (defined as the square of the standard deviation over the mean for a given image’s pixel intensities), the sample is in the optimal bright-spot plane in terms of image contrast. The sample can also be brought into focus for bright-field imaging (and Raman spectroscopy) by finding the local minima of variance closest to the bright-spot plane. This variance response for a given range of focal positions is shown in Figure 4.

**Figure A1:**
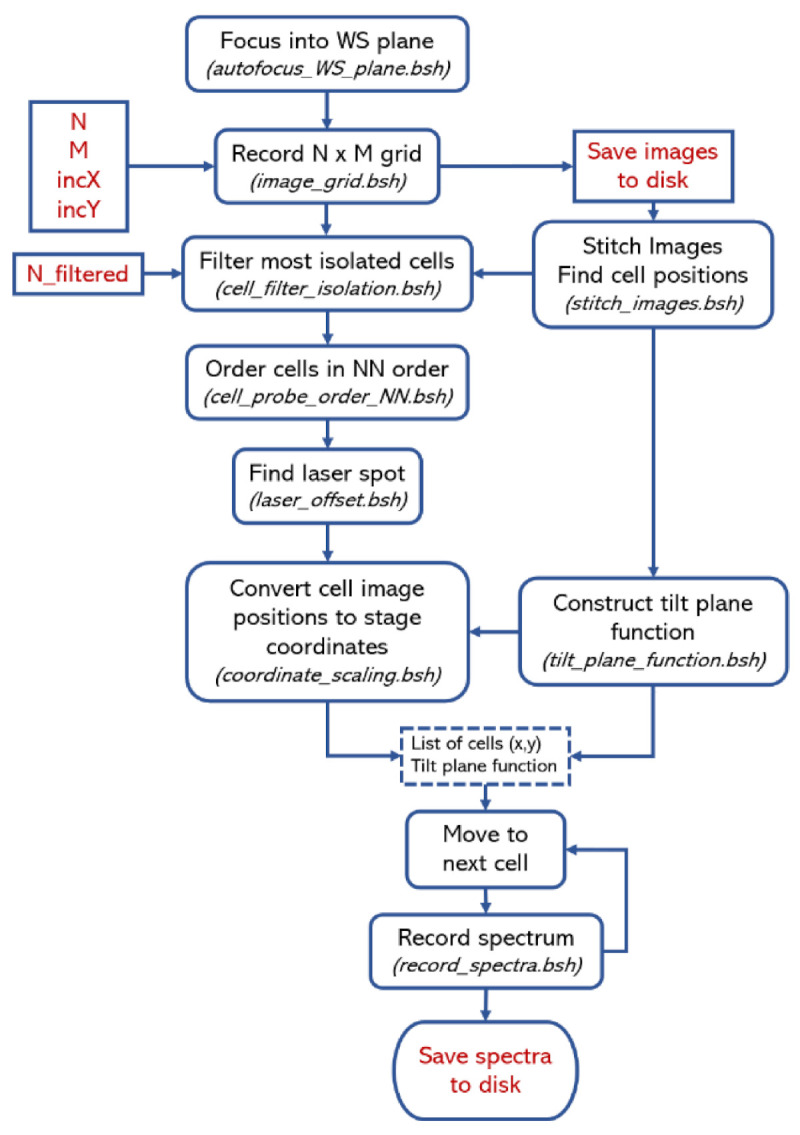
Flow chart of automated Raman spectroscopy routine. Flow chart detailing full list of processes to automate cell detection and probing. Manually set global variable inputs, and resultant output data highlighted in red.

The overall automation routine is illustrated in the flow-chart in Figure 4 with a series of high-level steps. A more comprehensive low-level flow-chart Figure A1 and description of the script logic is provided in the Appendix A, which corresponds directly with the source code provided in the online repository [26]. Once the bright-spot plane position is determined, it is relatively simple to acquire the positions of thousands of cells. A grid of overlapping images are recorded and stitched together, using built-in ImageJ plugins. ImageJ analysis tools also allow for local maxima present within the resultant image to be found and listed.

This list of coordinates includes thousands of cell nucleus positions, which can be further refined. Of these, a preset amount of the most isolated cells are sorted and filtered, removing the most clustered cells. By filtering for isolated cells, we improve the quality of the recorded spectra with respect to the targeting accuracy of the cell nucleus; as shown in the previous section, such clustered cells are more likely to be doubly targeted, particularly for the case of ThinPrep slides. The refined list is then sorted, using a nearest neighbour algorithm to limit stage movement, reducing the risk of drift and also speeding-up the overall acquisition process. Information from the image stitching process about the image overlap relative to the stage movement is used to create a coordinate transform, converting the list of cell positions from pixel coordinates to stage positions, which is described in more detail in the Supplementary Information. Once an offset for the laser spot position is included, and the stage is moved into the focus plane, the cells can be targeted by the Raman laser and the spectra can be recorded. The final step in the process is the removal of the baseline and glass spectrum component in these spectra as described in more detail in Section 3.5.

## 3. Materials and Methods

### 3.1. Sample Preparation

#### 3.1.1. HeLa Cell Culture for Fluorescence vs. White-Spot Comparison

HeLa cells were cultured in DMEM media (Sigma-Aldrich, St. Louis, MO, USA) supplemented with 5% fetal bovine serum. Flasks were maintained in a humidified environment at 37 ${}^{{}^{\circ}}$C and 5% CO${}_{2}$. When the cells reached 90% confluency, the culture media was removed and the cells were washed with sterile PBS. Trypsin-EDTA solution (0.25%) was added and the cells were incubated at 37 ${}^{{}^{\circ}}$C for 5 min until the cells detached. DMEM media was added to the flask to neutralise the trypsin, and the entire contents were transferred to a sterile falcon tube. The cells were centrifuged for 5 min at 1500 rpm. The supernatant was removed, and the cell pellet was resuspended in fresh DMEM media. The cells were counted, using a haemocytometer. After counting the cells, the cells were prepared for live cell imaging, and using the ThinPrep standard: (1) For the live cell study, the HeLa cells were seeded at $1\times 10^{5}$ cells/mL in a 12-well cell tissue plate. The next day, when the cells had reached 70% confluency, 2 drops of NucBlue™ Live ReadyProbes™ Hoechst 33342 (Thermo-Fisher, Waltham, MA, USA) were added to each well. The cells were incubated for 20 min before imaging. (2) For ThinPrep, 500,000 cells were transferred to a new falcon tube which was centrifuged at 1500 rpm for 5 min. The cell pellet was resuspended in 20 mL of PreservCyt (Hologic, Marlborough, MA, USA) and incubated for 20 min at room temperature. The cell suspension was loaded into the ThinPrep 2000 (Hologic, Marlborough, MA, USA) machine, and the cells were transferred onto a glass slide. No coverslips were applied.

#### 3.1.2. Bladder Cancer Cell Lines for Automated Raman Cytology

High (HT1197; ATCC) and low (RT112, Sigma-Aldrich) grade urinary bladder carcinoma epithelium cells were cultured in 1:1 mixture of DMEM and Hams-F12 medium supplemented with 5% fetal bovine serum and 2 mM LGlutamine. Flasks were maintained in a humidified atmosphere with 5% CO${}_{2}$ at 37 ${}^{{}^{\circ}}$C. When the cells reached 80% confluency, the culture medium was removed, and the cells were rinsed with sterile PBS. Trypsin-EDTA (0.5%) was added to the flask, which was incubated at 37 ${}^{{}^{\circ}}$C until the cells had detached. An equal volume of 5% serum-containing medium was added to the flask to neutralise the trypsin enzyme. The cells were collected in medium and transferred into a sterile container, and centrifuged at 1200 rpm for 5 min. The supernatant was removed, and the cell pellet was resuspended in fresh medium. This solution was centrifuged at 1200 rpm for 5 min, and the medium was decanted and resuspended in 1 mL PBS. This step was repeated and the cell pellets were resuspended into a vial containing 20 mL of a methanol-based fixative (PreservCyt; Hologic, Marlborough, MA, USA) and left at room temperature for 15 min. The vial was inserted into a ThinPrep 2000 (T2; Hologic, Marlborough, MA, USA) machine, and the cells were transferred to a CaF${}_{2}$ (Raman Grade; Crystran, UK) slide, or a glass slide (ThinPrep slide; Hologic, Marlborough, MA, USA). No coverslips were applied.

### 3.2. Fluorescence Microscopy

Fluorescence images were recorded, using an IX81 microscope attached to a MT20 illumination system with a 150 W Xenon arc burner and a filter set, providing excitation at 358 nm and emission at 461 nm. Images were recorded on a low-noise CCD detector (QIClick™, QImaging, UK) and an Olympus LUCPlanFLN 40X/0.6NA microscope objective was used to record the images, with a variable coverslip correction of 0–2 mm. Fluorescence images were captured with an acquisition time of 0.5 s, and matching bright-field images were recorded with an acquisition time of 0.02 s. For the case of the live cells, the coverslip correction was set to 1 mm, while for ThinPrep, it was set to 0 mm.

### 3.3. Automated Raman Optical System

Raman spectra from the cells described in Section 3.1 were recorded, using a custom-built Raman micro-spectroscopy system, which is illustrated in Figure 3. This system employs a 150 mW laser with a wavelength of 532 nm and a coherence length of >100 m (Torus, Laser Quantum), which is driven by a power supply unit (mpc3000, Laser Quantum) that is controllable over an RS232 cable, using serial commands using the Micro-Manager ‘freeserialport’ device adapter. The system also employs a spectrograph (Kaiser, Holospec f/1.8i), operating with a 25 μm slit and a holographic grating (HSG-532-LF). The spectrum is recorded, using a low-noise cooled CCD camera (**CCCD**, DU920P-BEX2-DD, Andor, Belfast, Northern Ireland) with 1024 × 256 pixels, of size 26 × 26 μm cooled to −80 ${}^{{}^{\circ}}$C and operating in full-vertical-bin mode. In this configuration, the camera provided a read noise standard deviation of 4 electrons/spectral sample, and a mean dark current of 0.0512 electrons/second/spectral sample. The camera was controlled, using the Micro-Manager Andor device adapter. Together, the spectrograph and CCD provided a bandwidth of -34-2517 cm${}^{-1}$ and an average resolution of 5.48 cm${}^{-1}$. Included in the spectrograph housing was a holographic notch filter (Kaiser; HSPF-532.0), providing an optical density of 6 at the laser wavelength and a spectral bandwidth of 350 cm${}^{-1}$. The laser and spectrograph were coupled into a fully automated inverted microscope (IX81, Olympus, Tokyo, Japan) controlled via the IX2-UCB control box, which allows for electronic control of the objective focus position (**Z**) and the white-light halogen–tungsten lamp (**HL**). This control box could be controlled, using an RS-232 cable, using the Micro-Manager Olympus IX81 device adapter. The microscope included a closed-loop high precision stepper motor translation stage (**XY**, 96S108-O3-LE, Ludl, Hawthorne, NY, USA) with a linear encoder, which provides repeatability of 0.25 μm and a resolution of 100 nm. The stage was driven by a control system (MAC 5000, Ludl), which could be controlled using an RS-232 cable, using the Micro-Manager Ludl device adapter. A 40× microscope objective, with numerical aperture of 0.75 (UMPlanFl, Olympus), was used to image the spectral irradiance to a 100 μm confocal aperture (**CA**, P100D, Thorlabs, Newton, NJ, USA), which isolated the signal from the cell nucleus in three dimensions, and minimised background noise from the glass slides, as well as from optical elements in the system. The system was designed to provide a spatial resolution of ≈2–3 μm${}^{2}$ from the cell nucleus. A long pass filter (**F**, LP03-532RU-25, Semrock) and a dichroic beamsplitter (**DB1**, LPD-01-532RS, Semrock, NY, USA) were also used to filter the laser wavelength from reaching the spectrograph, while transmitting the longer Raman scattered wavelengths. A dichroic short pass filter (**DB1**, 69-202, Edmund Optic, Barrington, NJ, USA) permits imaging of the sample to a digital camera (**CMOS**, MU300, AmScope, Irvine, CA, USA). All spectra were recorded using the Andor Solis software plugin for Micro-Manager. The system was wave-number calibrated, using a polymer standard as described in [33]. No intensity calibration was performed for this experiment since all spectra were recorded from the same system. The aperture in the microscope condenser (**C**, U-UCD8, Olympus) was closed to a minimum in order to maximise the spatial coherence of the illumination.

### 3.4. Spectral Acquisition

The automation process described in Section 2 is capable of recording a large number of spectra. In total, spectra were recorded from 577 different HT1197 cell nuclei deposited on CaF${}_{2}$ (for the purpose of removing the glass spectrum as described in the following section), 6426 different HT1197 cell nuclei deposited on glass, and 7499 different RT112 cell nuclei deposited on glass. An acquisition time of 10 s was used to record a single spectrum from within the nucleus of each cell. In addition, 20 spectra were recorded from the ThinPrep glass substrates, each with a 30 s acquisition time, which is required during pre-processing as described below. Each recorded raw spectrum contained 1024 samples in the range -34-2517 cm${}^{-1}$, from which 451 samples were extracted for further inspection, corresponding to the fingerprint region 600–1800 cm${}^{-1}$.

### 3.5. Pre-Processing of Raman Spectra

Raw spectra were first input to a cosmic ray removal algorithm [34]; this algorithm is capable of removing cosmic rays from a spectrum by identifying a closely matching spectrum in the dataset, and replacing each cosmic ray with the corresponding wave-number intensity values in that matching spectrum. It is, therefore, not necessary to apply the commonly used double acquisition cosmic ray removal method to obtain a closely matching spectrum [35], which was found to be problematic for the high-speed spectral acquisition applied in the automation process.

Cosmic ray removal was followed by an extended multiplicative signal correction (EMSC) algorithm [24,36,37], which removes the variable background signal from each spectrum. The EMSC algorithm estimates this background, using an N-order polynomial (to remove the baseline signal that results from the cells auto-fluorescence) and also, if required, the background signal from the substrate. Briefly described, the EMSC algorithm applies a least squares fit to (i) a low-noise, contaminant-free reference Raman spectrum from a cell; (ii) an N-order polynomial; and (iii) a reference spectrum taken from the substrate. This is required for the case of glass substrates but not for the case of Raman-grade CaF${}_{2}$. The algorithm returns the weight of (i), which enables normalisation of the spectrum relative to the reference, as well as the total background made up of the appropriately weighted substrate spectrum plus the polynomial. The EMSC-corrected spectrum, *X*, is given by the following:

(1) $$X=\frac{X_{0}-\left[B\times c_{b}\right]-\sum_{m=0}^{N}c_{m}P^{m}}{c_{r}}$$

where $X_{0}$ is the raw data, *B* is the reference spectrum of the substrate, $c_{b}$ is the weight of the reference substrate spectrum, $P^{m}$ denotes the $m^{th}$ order of the polynomial, $c_{m}$ is the corresponding polynomial coefficient, and $c_{r}$ is the weight of the cell reference spectrum, *R*. In summary, $X_{0}$ can be described as the linear (weighted) superposition of *R*, *B*, and *P*. It was shown that the use of a high-order polynomial does not result in over-fitting with the EMSC algorithm [37]. For this study, a fifth-order polynomial was used in the EMSC-correction algorithm for all datasets.

The reference cell spectrum provides the basis for all of the spectra to be fitted; the reference spectrum used here is the mean spectrum of the highest quality 50 spectra from the HT1197 dataset recorded on the CaF${}_{2}$ substrate. No smoothing was applied in this case. The background signal from Raman-grade CaF${}_{2}$ substrates are flat in the fingerprint region [38] and, therefore, this substrate was selected to obtain the high-quality reference cell spectrum to be used in the EMSC correction of the spectra recorded on the glass substrates. In order to remove any potential bias, the same reference spectrum was used for the EMSC algorithm applied to process the spectra of all cells deposited on glass. It was demonstrated that equivalent results, in terms of the multivariate statistical analysis that follows, will be obtained when using significantly different reference spectra, so long as the same reference spectrum is applied in EMSC correction of all datasets [24]. In addition, input to the EMSC correction algorithm is a glass spectrum, which is the mean spectrum of the spectra recorded from the substrate followed by Savitsky–Golay smoothing, using a polynomial order of 3 and a window size of 9.

Following EMSC correction, the spectra were denoised, using a Savitsky–Golay-based algorithm [39], using a polynomial order of 3 and a window size of 7. The resulting datasets of cell spectra were filtered in order to remove lower-quality spectra. This was achieved by removing all spectra that provided a Pearson correlation coefficient of less than 0.99 with respect to the reference cell spectrum, which resulted in a culling of approximately 50% of the data. A similar approach was previous applied, albeit with a lower coefficient value, in order to extract spectra with high signal-to-noise ratios from a large dataset [15]. The results of the various pre-processing steps described in this section are presented in Section 4.

### 3.6. Multivariate Statistical Classification

In order to comprehensively evaluate the capability of the automated system to accurately classify the low- and high-grade urinary bladder carcinoma epithelium cells, and to elucidate the underpinning differences in their biochemical composition, a range of machine-learning classification techniques were tested to discriminate the two spectral datasets, following the pre-processing steps outlined in Section 3.5. The algorithms considered were as follows: linear discriminant analysis (LDA), quadratic discriminant analysis (QDA), k-nearest neighbours (kNN), random forest (RF) [40], support vector machine (SVM) [41] and partial least squares (PLS) [42]. The classifiers were combined with two pre-processing steps, namely, principal component analysis (PCA) [43] and marginal relevance (MR) for wavelength selection [44]. PCA obtains lower dimensional projections of the data in the feature space. The new features (principal components—PCs) represent directions in the observation space along which the data have the highest variability. In contrast, marginal relevance (MR) is a criterion that ranks each wavelength in order of their capability to discriminate between the classes. The MR score for each wavelength is the ratio of the between-class to within-class sum of squares. In this approach, each wavelength is considered independently of others, and neighbouring wavelengths have similar MR scores.

All of the analysis was done in **R**, a free software environment for statistical computing and graphics [45]. LDA and QDA were implemented using the **MASS** [46] package and required no parameter tuning. PLS was implemented in the package **pls** [47], and the number of latent variables was set to 15. This parameter was set at a large constant value rather than being optimised using further tuning and cross-validation schemes. SVM was implemented in the package **kernlab** [48]. Gaussian kernel was used with the bandwidth parameter value set at an empirical estimate suggested by [49]. kNN and RFs were implemented in the packages **class** [46] and **ranger** [50], respectively. In RF, the number of trees was set at 500, and the number of variables to possibly split at in each node was set at the rounded down square root of the number of wavelengths. The number of neighbours for kNN was set at five.

Ten-fold cross-validation was used to estimate the performance of the models on new data. The folds were stratified within the class using **R** package **caret** [51]. PCA and MR preprocessing steps were carried out on the training set of each cross-validatory split of the data. Three PCs were used as input features to LDA, QDA and kNN classifiers. A marginal relevance (MR) criterion was implemented in the **R** package **BKPC** [52]. The wavelengths with highest MR scores from ten regions were taken as inputs into the following classification algorithms: LDA, QDA, kNN, RF and SVM. RF, SVM and PLS were also trained on all wavelengths without any dimension reduction pre-processing steps.

## 4. Results

**Figure 5:**
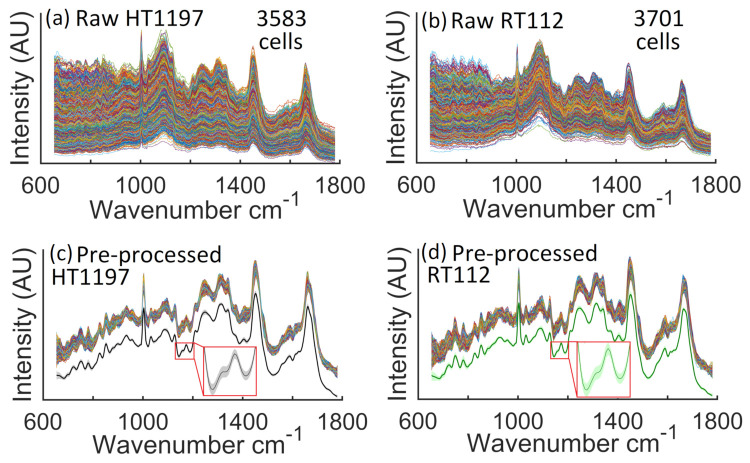
Raw and processed data from high- and low-grade bladder cancer cell lines. Raw spectra recorded using the proposed automated Raman micro-spectrometer, following cosmic ray removal; (**a**) 3583 spectra taken from individual HT1197 cell nuclei; and (**b**) 3701 spectra taken from individual RT112 cell nuclei; (**c**) the pre-processed HT1197 dataset, below which the mean spectrum is shown with a black line around which the standard deviation of the dataset is shown using a shaded grey colour; (**d**) the pre-processed RT112 dataset, below which the mean spectrum is shown with a green line around which the standard deviation of the dataset is shown using a shaded light green colour.

Following application of the Pearson correlation coefficient, the two spectral datasets of 6426 HT1197 (high-grade bladder cancer) cell spectra, and 7499 RT112 (low-grade bladder cancer) cell spectra, were reduced in number to 3583 and 3701 cell spectra, respectively. This corresponds to the retention of 56% and 49%, respectively. The raw spectra corresponding to these two culled datasets are shown in Figure 5a,b, following cosmic rays removal. It is clear from the figure that there are significant differences across the dataset of raw spectra; this includes a variation in the minimum value of the different spectra resulting from changes in the camera temperature throughout the experiments, which affects the mean camera dark current from one recording to the next. Additionally noticeable is a difference across the dataset in the relative amplitude of the various spectra, which results from slight variation in the focussing of the MO from cell to cell as well differences in the morphology of the cell nuclei. In Figure 5c,d the same two datasets are shown following pre-processing to remove the slightly varying baseline, and the glass spectrum as described in Section 2.2. Additionally shown in these figures are the mean spectra of the pre-processed datasets as well as the standard deviation of the pre-processed datasets around this mean value. It is clear from these results that the glass signal, most present in the raw spectra within the 1050–1150 cm${}^{-1}$ region is reduced significantly.

**Figure 6:**
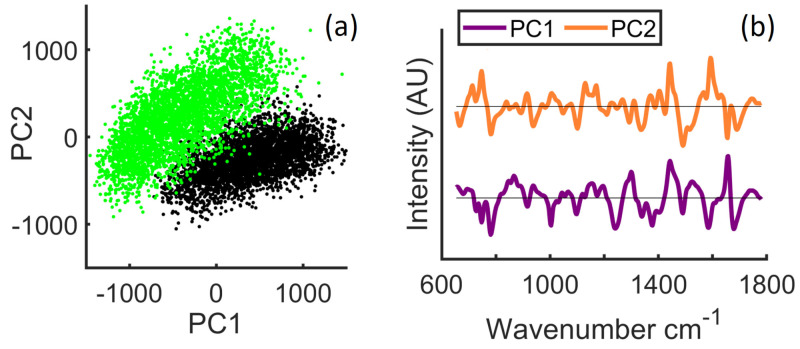
Multivariate statistical analysis: (**a**) PCA scatter plot; (**b**) first two principal components.

In Figure 6, the results of principal components analysis applied to the two pre-processed datasets are shown. In Figure 6a, a scatter plot is shown for the scores of the first two principal components, and in Figure 6b, the corresponding loadings are shown. The explained variance for the first two principal components, PC1 and PC2, are 35.97% and 15.17%, respectively. It is clear that the two datasets separate well over these first two components. Inspection of these two principal components revealed several peaks that were previously identified in the study of bladder cancer with Raman spectroscopy: 680 cm${}^{-1}$ (DNA), 789 cm${}^{-1}$ (DNA), 1003 cm${}^{-1}$ (phenylalanine), 1170 cm${}^{-1}$ (tyrosine), 1303 cm${}^{-1}$ (CH${}_{3}$, CH${}_{2}$, lipids, DNA), 1370 cm${}^{-1}$ (DNA, lipids), 1220– 1300 cm${}^{-1}$ (amide III, collagen) [6,53] as well as peaks at 1093 cm${}^{-1}$ (PO2 stretching, DNA/RNA), 750 cm${}^{-1}$ (tryptophan), 1310 cm${}^{-1}$ (guanine), 1340 cm${}^{-1}$ (adenine), and 1580 cm${}^{-1}$ (tryptophan, DNA, phenylalanine) [5,11,53], as well as two new peaks that were not previously identified in the analysis of bladder tissue—1424 cm${}^{-1}$ (deoxyribose) and 1490 cm${}^{-1}$ (DNA) [54].

**Figure 7:**
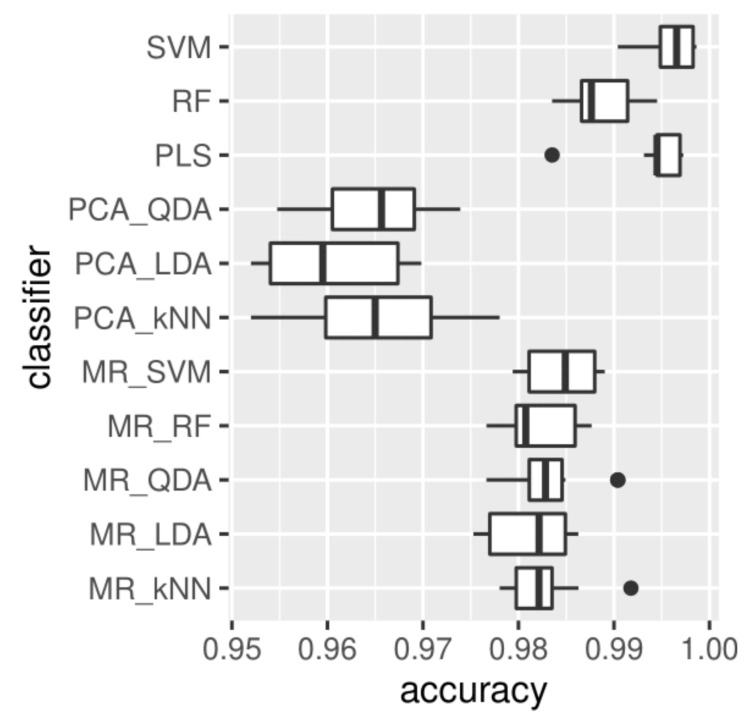
Classification accuracy of the 10 test sets in ten-fold cross-validation for the 11 classifiers.

Classification accuracy, sensitivity, and specificity for the 11 classification approaches are given in Table 1, and Figure 7 shows box-plots of classification accuracy over the test sets in ten-fold cross-validation for the 11 classifiers. The comparative analysis suggests that classifiers PLS, SVM, and RF, with no (statistical) pre-processing steps, consistently perform better for classification of these datasets. MR seems to be more effective than PCA, with three PCs in selecting the features. We note that these sensitivities and specificities are the highest reported to date in the literature in the classification of high- and low-grade bladder cancer cell lines, likely owing to the increased reproducibility of the recording process as well as the increased dataset size.

## 5. Discussion

The automated Raman cytology system presented here is focused on recording spectra from the unlabelled nucleus of epithelial cells. Automated targeting of the nucleus is a key differentiator of the proposed technology with other automated Raman cytology systems, which were recently proposed and which target the centre of the cell mass [15,16]. The basis of our approach is to iterate the focus of the microscope between two planes—the traditional image plane at which Raman spectra are recorded, and the ‘bright-spot plane’ some tens of micrometers away from the image plane, where the ‘micro-lens’ effect of the cell nuclei focuses the partially coherent microscope illumination and produces bright spots approximately co-located with the cell nuclei, thereby facilitating subsequent RS. Our approach builds on the work of Drey et al. [29], who first identified the phenomenon and utilised it for the purpose of cell counting of live cell cultures. The ‘focal-length’ of the nucleus, i.e., the distance between the microscope focal plane and the ‘bright-spot plane’, appears to depend on the spatial variation in cell thickness, with a thicker, rounder morphology resulting in the focusing of light several micrometers behind the sample plane, and a thinner, flatter morphology focusing the light several tens of micrometers behind the sample plane.

We have shown that in addition to live adherent epithelial cells, the method can also be applied to identify the nucleus of cells prepared using the ThinPrep standard, which includes the use of specific fixatives and glass slides. Appending such a widely established clinical practice for preparing uniform patient samples for subsequent pathological classification is an attractive means for advancing Raman cytology into the clinic as an assistive tool for cyto-histopathologists. HeLa cells prepared using ThinPrep, and compared with the same cells growing in medium, revealed a stark difference in morphology, with the former appearing significantly thicker and having a smaller footprint. Interestingly, this translated to a significantly shorter nucleus ‘focal-length’. Nevertheless, identifying the nucleus could be achieved for either method of cell preparation, which indicates that the proposed automated Raman cytology system could be applied both to growing cell lines for the purpose of basic research, as well as to patient cytology samples prepared using the ThinPrep standard. Over both methods of cell preparation, we found accurate nucleus identification for approximately 75% of cells. We believe this could be improved upon by using a colour filter in the illumination lamp to increase the bright-spot contrast as in [29], or for the case of live cells, to immerse the cells in phosphate buffered solution before imaging, which was shown in [29] to have the effect of swelling the cell body and significantly enhancing the bright-spot contrast.

The overall throughput of the method is demonstrated at approximately 0.1 cell/s, which is slower than the method proposed by Schie et al. [15], which can record at a rate of approximately 1.4 cell/s. However, this comparison must be qualified by the cell type that was investigated by the two systems. In [15], the authors applied their system to lymphocytes, neutrophils, and monocytes, which are considerably thicker than the epithelial cells investigated in this paper, and which, therefore, produce a more intense Raman scattering, necessitating a shorter acquisition time. Furthermore, they used a significantly more powerful laser source of 400 mW, albeit at a longer wavelength of 785 nm, which produces less Raman scattering [38]. Noticeably, they also used a Pearson correlation coefficient of 0.95 to cull their noisy spectra, while we used a coefficient value of 0.99. We found that an acquisition time of 5 s (increasing the throughput of 0.2 cells/s) could be used if we apply a value of 0.95. We believe that it may be possible to further reduce the acquisition time by increasing the laser power, distributed over a larger area of the cell nucleus, and using advanced denoising methods based on machine learning. With these approaches, we believe it may be possible to achieve 1 cell/s throughput, and this will be a subject of our future work.

We note that in this study, only a single pair of cell cultures was employed, corresponding to low and high grade bladder cancer. The central theme of the paper is to demonstrate the working principle of our automated Raman cytology system, which we believe is achieved, using a single set of BC cell cultures. Future application of the device in the classification of cell cultures should employ several replicate cell growths in order to better validate the results.

The cell nucleus is the key region of interest in Raman cytology. An important point of discussion is the degree to which a single (confocal) Raman spectrum from an area of 2–3 μm is representational of the whole of the cell nucleus. Spectra recorded from different spatially resolved points in a single cell nucleus can significantly differ should they originate from different sub-nuclear features, such as nucleoli [55]. We expect that the proposed system will produce superior classification accuracy if the spectrum can be recorded from a larger area of the nucleus, particularly for cases where there is a high degree of biochemical similarity between the cell lines under investigation. One solution is to increase the laser spot size as well as the confocal aperture size. However, this approach will reduce the depth selectivity of the confocal aperture and has the potential to significantly increase the contribution of the glass in the spectrum; glass is unavoidable for clinical applications. Another solution involves the recording of spectra from several points in the nucleus, which are then averaged to produce a single representational cell nucleus spectrum [56]. A related solution is to move the cell during a single acquisition time such that various points in the nucleus are scanned over the laser spot. Enhancing the proposed system to produce a more representational cell spectrum is an important direction for future research.

Although we have demonstrated the applicability of the automated identification of the nucleus for both live cells and cells prepared using ThinPrep, we focus our experiments in Section 4 on bladder cancer cell lines using the latter. The application of the proposed system to clinical cytology samples is the primary motivation of our work. An important feature of our automated platform is the use of 532 nm excitation and the removal of the glass spectrum, which is unavoidable for clinical deployment of the technology. We have identified at least three clinical areas that could potentially benefit from the proposed technology, all of which were shown to be diagnostically improved by Raman spectroscopy. The most common branch of cytology is the ‘Pap smear,’ used to screen precancerous cervical lesions; application of Raman to cervical cytology samples has received significant attention in the literature [7,11,12,13]. Cytological inspection is also common for bladder cancer, whereby epithelial cells are extracted from urine, though this is often used as an adjunct to cystoscopy, due to the low sensitivity (20%) for low grade carcinoma accounting for the majority of cases. RMS was demonstrated to improve the sensitivity of urine cytology to >90% [3,4,6] and our group actively researched a methodology for RMS to be integrated into the pathology lab [5]. Oral cancer is one of the most common cancers worldwide, with tumours being located around the tongue and mouth. Late stage oral cancer is straightforward to diagnose with histological analysis of tissue biopsy, but results in poor outcomes. RMS was demonstrated to successfully identify precancerous tissue by investigating epithelial cells [8,14]. All three of these clinical areas require only non-invasive procedures to retrieve cells, and although each of them benefit from improved classification using RMS, clinical adoption has been slow, due to the slow throughput of Raman and issues with reproducibility. We believe that the system proposed here can solve these issues and facilitate clinical adoption.

A final point of note in this discussion is the development of the proposed automation routine, using the Micro-Manager platform. This software is open-source and readily applicable to any existing electronically controllable microscope and RMS systems that are commercially available. The programming scripts that we have developed are provided in an online repository [26] and can be downloaded and adapted with relatively little effort. We hope that this approach will help to advance automated Raman cytology for clinical applications.

## 6. Conclusions

In this paper, we have demonstrated an automated Raman cytology system that is capable of targeting epithelial cell nuclei for the primary purpose of improving the throughput and reproducibility of the clinical application of Raman cytology. Importantly, the system can be applied to both living adherent epithelial cells in medium for basic research, as well as cells prepared using the ThinPrep standard, an established cell fixation and deposition protocol for cervical, urine, and oral cytology, which were all previously shown to be enhanced by Raman spectroscopy. Using two clinically relevant cell lines—high- and low-grade bladder cancer cell lines—we demonstrated a cell throughput of 0.1 cell/sec and discussed approaches for further increasing this. The automation routine was developed using the open-source Micro-Manager software system and can readily be downloaded and adapted for existing RMS systems. We hope that this work will provide the much-needed throughput and reproducibility to finally advance Raman cytology into routine clinical practice.

## Figures and Tables

**Table 1:** Classification accuracy, sensitivity, and specificity for the 11 classifiers: LDA, QDA and kNN applied after pre-processing with PCA and MR. RF and SVM were trained after pre-processing with MR and on the entire data without any pre-processing for dimension reduction. PLS was applied without any pre-processing.

	Accuracy	Sensitivity	Specificity
SVM	0.996	0.996	0.996
RF	0.989	0.989	0.988
PLS	0.994	0.995	0.994
PCA_LDA	0.960	0.952	0.969
PCA_QDA	0.964	0.962	0.966
PCA_kNN	0.966	0.959	0.972
MR_LDA	0.981	0.985	0.978
MR_QDA	0.983	0.982	0.985
MR_kNN	0.983	0.980	0.986
MR_RF	0.982	0.984	0.980
MR_SVM	0.984	0.986	0.983

## Data Availability

All automation code is available from a publicly accessible repository [26]. Spectral datasets are available on request from the corresponding author.

## Appendix A. Raman Automation Scheme

The automation scripts described in the main text are designed to receive slides of fixed, isolated cells, locate their positions with a high degree of accuracy and probe them with Raman spectroscopy for chemical analysis–based classification. The script then outputs a text file of spectra to be pre-processed followed by statistical processing, using a multivariate classifier, such as PCA-LDA. The full Beanshell scripts for the automated Raman spectroscopy are provided at [26]. These scripts are referenced throughout the discussion below and in the detailed flow chart shown in Figure A1.

The main script calls a series of scripts that perform discrete functions for the overall scheme. This is to allow for ease of use with respect to modification and the addition of further functionality in future iterations. The following will explain the purpose of each script and necessary considerations for adapting them to different platforms and/or applications. While the scheme is designed to be as system agnostic as possible, where decisions are made to account for specific system configurations, it will be noted.

The main script “1_objective_automation.bsh” calls all subsequent scripts, imports the necessary ImageJ routines, initialises the hardware of the microscope, laser and both cameras and provides the necessary global variables. The script first calls the autofocus routine for the bright-spot plane, “autofocus_WS_plane.bsh”. The script assumes that the microscope stage is at or near the focal plane of the sample. The script scans a range of 300 μm to find the bright-spot plane. A stack of 60 images are recorded with the imaging camera and saved to disk. ImageJ functions open the image stack and perform the variance calculations to determine the correct plane of interest.

The next script, “image_grid.bsh” records a grid of overlapping images, from which the overall field-of-view is constructed. The size of the grid, as well as the increment of stage movement between them, is manually determined to account for the camera aperture, objective magnification and stage control. As such, they are defined as global parameters set by the main script. They are similarly saved to disk. A separate script, “stitch_images.bsh”, opens the images and performs the stitching process via ImageJ. The calculation here is time intensive, as the overlap between images is not assumed to account for changes in the microscope system. The images are blended, so discontinuities do not create artefacts, which might interfere with finding the cell positions. The ImageJ process “Find Maxima” is sufficient to collect a list of bright-spot coordinates, which are taken to be the cell positions. Acquisition times, lamp light levels, and condenser annulus alignments are all made with direct consideration for the resultant image produced and processed by this script. The stitching plugin also produces a text file which contains information necessary for “coordinate_scaling.bsh”.

The nucleus “micro-lens effect” is found to produce the most accurate positions of the cell nucleus when the cell is in isolation. For this reason, the most isolated cell are prioritised. The script “cell_filter_isolation.bsh” sorts the list of coordinates by their proximity to the closest neighbouring coordinate, and selects the most isolated cells first. This script filters so the output is a fixed number of cells, set as a global variable. This is done to standardise the recording times. This script could be modified to set a minimum threshold of cell separation or some fixed fraction of available cells, if upper limits on recording time is not a consideration. The script “cell_probe_order_NN.bsh” orders the given list of coordinates with a nearest neighbour algorithm to limit excessive stage motion.

The second autofocus script “autofocus_image_focus_plane.bsh” focuses into the in-focus plane for bright-field imaging. As the cells are largely transparent, the focal plane exhibits a local minimum in the image variance as a function of the focal depth. This script moves in smaller steps with an overall range of 20 μm. This script only finds the correct plane if the Z-stage is within 10 μm of the plane. As best practice, the bright-spot plane autofocus script is used before this one in order to ensure the best results.

The stage must be in the image focus plane for use of the Raman laser. The position of the laser spot on the imaging camera, found by “laser_offset.bsh”, is necessary to move the cells directly in the path of the laser. The laser is set to minimum power as to not damage the imaging camera.

The list of cell positions must now be converted from pixel coordinates into values usable by the stage. This is implemented by means of a rotation-translation-scaling coordinate transform, using calculations rather than fixed ratios in order to ensure applicability across different microscopes, objective lenses, cameras, etc. The displacement in pixels between subsequent images in “stitch_images.bsh” is related to the fixed movements driven by the stage to calculate the transform. The output of this script provides the list of cell coordinates to be driven by the stage.

“tilt_plane_function.bsh” is an error correction for focal drift should the sample not be level on the stage. The final script “record_spectra.bsh” handles the moving and recording of the Raman spectra for the cells, and saves the spectra to disk. These raw data can then be processed for statistical analysis.
